# ETHER: a proposal for a European peri-operative data sharing and registry network

**DOI:** 10.1097/EJA.0000000000002366

**Published:** 2026-02-25

**Authors:** Wilton A. van Klei, Claudia Spies, Falk von Dinklage, Jona Joachim, Michel MRF Struys, Michelle S. Chew, Olegs Sabelnikovs, Oscar Díaz-Cambronero, Shruthi Madappa, Wolfgang F. Buhre

**Affiliations:** From the Department of Anaesthesiology and Intensive Care Medicine, University Medical Center Utrecht, Utrecht, The Netherlands (WAVK), Department of Anaesthesiology and Intensive Care Medicine, Charité- Universitätsmedizin Berlin, Berlin (CS), Department of Anesthesia, Intensive Care, Emergency and Pain Medicine, University Medicine Greifswald, Greifswald, Germany (FVD), Department of Anaesthesiology and Intensive Care Medicine, AP-HP, Hôpital Lariboisière, Paris, France (JJA), Department of Anaesthesiology, University Medical Center Groningen, Groningen, The Netherlands (MMS), Division of Anaesthesiology and Intensive Care, Department of Peri-operative Medicine and Intensive Care, CLINTEC, Karolinska Institute, Stockholm, Sweden (MSC), Department of Anaesthesiology, Intensive Care and Clinical Simulations, Riga Stradins University Hospital, Riga, Latvia (OS), Department of Anaesthesiology and Intensive Care, Hospital la Fe, Valencia, Spain (ODC), Department of research, European Society of Anaesthesiology and Intensive Care, Brussels, Belgium (SM) and Department of Anaesthesiology and Intensive Care Medicine, University Medical Center Utrecht, Utrecht, The Netherlands (WFB)

## Abstract

Peri-operative medicine is a critical component of contemporary healthcare delivery. Despite significant advancements, peri-operative complications remain a relevant concern. Obtaining reliable risk estimates, identifying potential causes, and studying new interventions, revised policies or implementation of best practices to prevent complications, requires data from a large number of participants. Electronic Patient Record systems offer the opportunity to unlock these data, but the limited standardisation of databases and sharing frameworks available across Europe limit the effective use of the available data.

We propose creating a European peri-operative shared data registry with continuous data collection, integrating clinical, bedside monitoring and outcome data in a collaborative network. Such network would facilitate outcomes research, could serve as a platform to optimise clinical practices by fostering quality improvement through benchmarking of care delivered by departments or individual physicians, and could be used to evaluate policy changes. This ESAIC initiative aligns well with the development of the European Health Data Space.

This article provides examples of contemporary clinical research and practice evaluation questions to illustrate the need for a European collaborative data-sharing network, highlights inspiring examples of existing data-sharing initiatives and describes a road map to establish such network.

## The need for a European peri-operative data sharing and registry network

Peri-operative medicine, encompassing the co-ordinated care of patients before, during and after surgery or other invasive interventions, is a critical component of contemporary healthcare delivery. Despite significant advancements in peri-operative medicine over the past decades, complications remain a relevant concern as they significantly contribute to increased morbidity, mortality, and costs. Moreover, rare peri-operative events may go unrecognised, yet still adversely affect long-term healthy aging. Obtaining reliable risk estimates, identifying potential causes of adverse events, and studying new interventions, revised policies or (implementation of) best practices to prevent these peri-operative complications, requires data from a large number of participants. Such data could serve sophisticated, data-driven assessment of risks and benefit of interventions and will facilitate shared decision-making throughout the entire peri-operative pathway.

In today‘s era, Electronic Patient Record (EPR) systems offer the opportunity to unlock data from an almost unlimited number of patients, a development strongly encouraged by health policymakers for use in research, evidence-based decision-making and quality improvement. Data-driven and artificial intelligence-based applications may improve the delivery of individualised care and address potential future shortages in healthcare personnel. Although Europe is densely populated, and EPR systems are being increasingly adopted, the limited standardisation of health record databases and sharing frameworks available across Europe significantly limits the effective use of the available patient data in research.

To address this gap, we propose creating a European peri-operative shared data registry with continuous data collection, integrating clinical, bedside monitoring and postoperative outcome data. This initiative, under the auspices of the European Society of Anaesthesiology and Intensive Care (ESAIC), includes experts in peri-operative medicine, anaesthesia, intensive care and data science from several European countries. We will bring together researchers, healthcare institutions, technology providers and industry partners to establish a collaborative network for data collection, sharing, analysis, and dissemination of results. In addition to facilitating outcomes research, European multi-institutional sharing of peri-operative data could also serve as a platform to optimise clinical practices by fostering quality improvement and enabling benchmarking of care delivered by departments or individual physicians, and for evaluating new policies or policy changes. Our initiative aligns well with the current development of the European Health Data Space and aims to provide a peri-operative use case for this broader European vision.

**Fig. 1:**
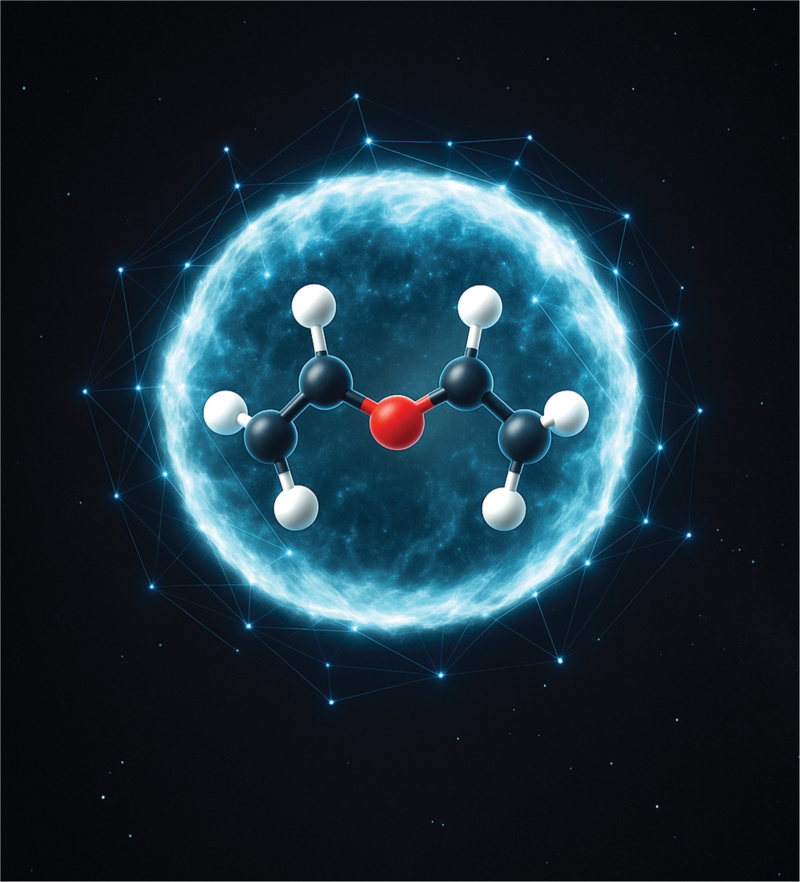
Logo for ETHER, the European peri-operative datasharing and registry network.

To illustrate the need for a peri-operative data sharing and registry ETHER network (Fig. 1), we provide several examples of contemporary clinical research and practice evaluation questions. In addition, we will highlight some inspiring examples of existing data-sharing initiatives. Finally, we describe a road map to establish such a collaborative European network, including expected challenges.

## Illustrative examples

Multicentre clinical databases are required for robust estimation of peri-operative risks, for efficient execution of randomised controlled clinical trials at reduced costs, and for benchmarking across populations. Obtaining reliable risk estimates and identifying potential causes of rare (incidence 1 per 10 000 or less), but devastating events, including those in subgroups, requires data from a large population. For instance, detecting a four-fold increase in incidence of a severe outcome with a baseline incidence of around 1 in 10 000, for example, mortality in patients otherwise deemed healthy, would require a sample size of roughly 100 000 subjects (assuming power of 80% and a two-sided alpha of 0.05). To detect a potentially still clinically relevant two-fold increase, the required sample size would rise to almost 600 000. There would be many such examples of the need for studying complications, (drug-related) side effects or outcomes of changes in practice in the peri-operative period. Without aiming to be exhaustive, we describe some examples of current questions that could be answered relatively easily if a collaborative European peri-operative data sharing and registry were available, including examples of prospective (quality improvement) research (Table 1).

**Table 1:** Illustrative examples demonstrating the need for a European peri-operative registry

Domain	Clinical question	Event/outcome	Why current data are insufficient	How the ETHER network helps
Drug safety	GLP-1 receptor agonists and risk of aspiration	Aspiration pneumonia	Very low baseline incidence and relatively low prevalence of exposed surgical patients in single centres or national datasets	Pooled multicentre European data enabling precise risk estimates, subgroup analyses and evaluation of mitigation strategies
Drug side-effects	Sugammadex or remimazolam-related anaphylaxis	Anaphylaxis, cardiovascular collapse, mortality	Fragmented reporting, under-ascertainment and heterogeneous definitions across institutions and countries	Standardised peri-operative phenotyping and cross-country signal detection within a harmonised data structure
Treatment failure	Accidental awareness with TIVA versus inhalational anaesthesia	Accidental awareness	Individual trials and single-centre datasets are underpowered for robust comparative effectiveness analyses	Large multicentre cohorts and the possibility of embedded pragmatic trials with common outcome definitions
Policy change	Implementation of fluid fasting policies	Aspiration pneumonia, patient comfort and satisfaction	Evidence mainly from single-centre experiences with limited power for harm and limited external validity	Rapid, multicentre evaluation of practice changes across different health systems and diverse patient populations
Personalised medicine	AI-based propofol dosing and haemodynamic management	Intra-operative hypotension, accidental awareness, quality of recovery	Limited datasets from highly selected populations and restricted monitoring modalities	Diverse, multinational registry incorporating detailed monitoring data to develop, calibrate and validate AI-based decision-support tools

AI, artificial intelligence.

### Complications

Glucagon-like peptide-1 receptor agonists (GLP-1-RA) are increasingly prescribed highly effective drugs for type 2 diabetes and obesity.^1,2^ They are associated with delayed gastric emptying and therefore anaesthesiologists are concerned about an increased aspiration and subsequent pneumonia risk in patients using these medications.^3–5^ Although patients on GLP-1-RA are generally considered to have an increased risk during general anaesthesia, reliable risk estimates for this feared complication are not available until now.^6,7^ The baseline risk of aspiration is low, as is the prevalence of GLP-1-RA use in surgical patients, but reported incidences vary considerably and the number of patients using GLP-1-RAs is increasing.^5,8,9^ Consequently, risk-mitigating measures advocated in current guidelines are not targeted and specific and thus may unnecessarily interfere with routine airway management in the operating room or with treatment of diabetes or weight control in many patients.^1,4,10^ Reliable risk estimates, including for subgroups, will improve individualised care for patients on these drugs.

### Drug side-effects

Peri-operative anaphylaxis is rare (estimated one to two cases per 10 000 procedures).^11,12^ Neuromuscular blocking agents, such as rocuronium, and antibiotics (e.g. cephalosporins) are the drugs traditionally implicated, yet high-quality epidemiological data and evidence-based management guidelines remain limited.^11–13^ It is relevant to mention that the incidence of mortality in patients experiencing peri-operative anaphylaxis is as high as 5%.^12^ In this regard, anaphylaxis after administration of remimazolam or sugammadex warrants urgent and focused attention.^14,15^ Both drugs are new and are increasingly being used. In particular, sugammadex can cause anaphylactic reactions during emergence from anaesthesia or even during transfer out of the operating room, potentially putting patients at high risk of severe consequences.^14^ Improved insight in the epidemiology and risk factors for drug-related anaphylaxis will improve peri-operative patient safety.

### Treatment failure

Intra-operative awareness is a serious and feared complication for patients, despite its low incidence (spontaneously reported one to two cases per 10 000 procedures).^16,17^ While patient factors (female sex, younger age, obesity), provider factors (anaesthetist experience) and surgical factors have been described, uncertainty remains as to whether total intravenous anaesthesia (TIVA), which is increasingly being adopted due to reported environmental benefits, affects awareness risk compared to inhalational anaesthesia.^16–19^ The ongoing large-sized (*n* = 13 000) THRIVE trial is conducted within a data-sharing consortium and specifically aims to answer this question.^20^ The THRIVE trial is a great example of embedding a potentially high-cost, and therefore difficult to fund, large-sized multicentre interventional study comparing two contemporary clinical practices within an established data-sharing platform, significantly reducing the efforts and costs of data collection.

### Embedded clinical trials

The recently published PRETREAT trial could serve as another example of embedding large-scale intervention studies in an ongoing registry.^21^ Using registry data to calculate the individual risk of intra-operative hypotension, PRETREAT randomised patients to different strategies for managing hypotension, stratified by individual risk. All data were routinely collected in daily care and retrieved from local data registries, resulting in the inclusion of over 3200 patients in 3 years with a relatively small budget of only €800 000. Such a trial could similarly be done at a low cost, but in much less time with more centres, if a multicentre European peri-operative shared data registry were available. This approach would also accelerate the necessary next steps in the process of unravelling the harms and benefits of (treating) intra-operative hypotension.

### Policy change evaluation

The need to be able to conduct fast, but adequately sized studies, assessing the effects of policy changes at low costs, can be further illustrated by another example. Although pre-operative guidelines for more than 20 years have advocated allowing patients to drink clear fluids up to 2 h before surgery, real-world implementation of these recommendations is poor.^22–24^ A quality improvement study performed in a Dutch hospital, therefore, estimated whether a rigorous and monitored introduction of a liberal fluid fasting policy, allowing for ingestion of clear fluids until arrival at the operating room, resulted in reduced fasting times without increased risk of aspiration.^8^ Conducted over 5 years and involving over 76 000 patients, fasting times for clear fluids indeed were significantly reduced to under 2 h. However, due to the rarity of aspiration, even this large sample was insufficient to definitively assess safety. Embedding such evaluations within a European registry would provide rapid, adequately powered assessments of policy changes at minimal cost.

### Quality improvement and personalised medicine

As a final example, we would like to mention a project integrating real-world patient data obtained from EPR into propofol pharmacological dosing models.^25^ Using machine learning techniques, this multicentre project aims to develop real-time automated algorithm-driven individualised dosing support within EPR systems, in order to improve depth of anaesthesia (less awareness) and reduce side effects (unintended hypotension).^26–28^ The result of this project aims to facilitate anaesthesia providers who currently dose propofol ‘manually’, without the need for target-controlled infusion pumps. The overarching future goal is to develop real-time dosing support for routinely used peri-operative drugs, combining pharmacological knowledge with available patient data. A large European peri-operative data-sharing network including data from patients with different ethnic backgrounds would facilitate such developments, also for medications that are used less frequently, and would facilitate harmonisation of diverse clinical practices.

The examples mentioned above and summarised in Table 1 seem relevant at this stage. Although research priorities will change over time and new critical issues will arise, the fundamental principle they illustrate, the necessity for a European peri-operative data-sharing network, remains unchanged. In an environment where undesirable outcomes are (fortunately) rare, such a collaborative approach is essential to accelerate evidence generation and improve patient safety. The overarching value of the proposed ETHER network, therefore, is in providing a flexible peri-operative data infrastructure capable of supporting a broad range of future clinical, quality and policy questions.

## Existing comparable data-sharing initiatives

Reducing the occurrence of undesirable peri-operative events through optimisation and harmonisation of unnecessary diverse clinical practices, and consequently improving quality of care, obviously can be achieved with benchmarking as well. In this, providing feedback to departments and individual providers based on shared data serves as a cornerstone. There are several comparable data-sharing initiatives to learn from for our proposal.

### The Multicentre Peri-operative Outcomes Group

The Multicentre Peri-operative Outcomes Group (MPOG) is an academic consortium representing over 80 hospitals, mainly from the United States, working together in a collaborative culture.^29^ Since its inception in 2008, MPOG has developed all the required preconditions to share, validate, and combine EPR and administrative data, in order to conduct large-scale quality improvement initiatives and research. According to their website, MPOG currently comprises over 28 million anaesthetic cases. Quality improvement is sponsored by the Blue Cross Blue Shield Association and is achieved by providing toolkits, individual feedback on chosen measures to anaesthesia providers in participating hospitals, and a reporting dashboard for benchmarking between hospitals. For research purposes, data are used to analyse the interplay between patient characteristics, surgical procedures, peri-operative care and postoperative outcomes. MPOG research priorities are collectively set by its members through a clinical research committee. In the past 15 years, over 80 scientific publications were based on MPOG data, of which 65 were from the last 5 years only, illustrating that it takes a long-term vision, perseverance, dedication and investments before harvesting from such an initiative.

### ICUdata4EU

This initiative, started in 2024, aims to standardise and improve the European cross-border use of patient data from critically ill individuals.^30^ Similar to MPOG's approach and our proposal for peri-operative data sharing, this initiative aims to improve clinical decision-making and personalised medicine in the intensive care environment by establishing a data infrastructure. It is funded via the European Union's Digital Europe Programme.^31^ ICUdata4EU brings together a diverse consortium of 18 partners from eight countries, including academic institutions, healthcare providers, technology companies, policy makers and patient advocates. According to the consortium website, the project is driven by well defined objectives. These include standardising data by creating unified formats and terminologies to eliminate inconsistencies and ensure seamless interoperability and data sharing across European ICUs. The ICUdata4EU initiative illustrates the importance of multidisciplinary collaboration, including both public and private partners.

### INDICATE

Connecting Data in European Intensive Care (INDICATE) aims to improve data sharing between ICUs across Europe.^32^ INDICATE is co-funded via the European Union's Digital Europe Programme.^32^ It focuses on developing and implementing trustworthy, inclusive, and diverse artificial intelligence models to improve individual patient care through ethically responsible data use. Interestingly, INDICATE will use a novel secure federated infrastructure, which sends analysis software to local data instead of transferring patient data to a centralised database. This ensures patient data remains within hospitals, hence safeguarding patient privacy by design. The INDICATE infrastructure example highlights solutions to comply with the General Data Protection Regulation directives.

Obviously, the ICU environment is different from the peri-operative environment, justifying our proposal for a separate European peri-operative data-sharing network. Although one can argue that investing in such a data-sharing platform next to MPOG is redundant, the difference between Europe and North America in healthcare systems, peri-operative care, available drugs and medical devices, in our opinion justifies the development of a separate European network.

## Road map

To achieve our 5-year goal of an established multi-institutional peri-operative federated data sharing and registry network within Europe, we have defined several intermediate steps as milestones (Table 2). Such a federated network has several requirements, for example, including a participation framework, a shared data model, and a secure platform. The requirements and expected challenges are described in more detail below.

**Table 2:** Proposed road map for establishing the ETHER network between 2026 and 2030

Milestones	Number of institutions	Funding	Type of data	2026	2027	2028	2029	2030
Partnership establishment with first two to three institutions	2 to 3	ESAIC + in-kind investment from participants	Intra-operative					
Federated data management infrastructure established	4 to 5	ESAIC + in-kind investment from participants	Full peri-operative					
At least one scientific publication	8 to 10	ESAIC + in-kind investment from participants, sustainable funding to be ensured until 2035	Full peri-operative including outcomes					
At least two scientific publications	12 to 15	Sustainable funding ensured until 2035	Full peri-operative including outcomes					
At least three scientific publications, initiation of a funded prospective study	20+	Sustainable funding ensured until 2035	Full peri-operative including outcomes					

### Partnerships

The success of a federated data network depends on a strong partnership strategy, which promotes wide engagement, shared vision, mutual benefit and long-term stability. The partnership initiation focuses on aligning objectives, defining roles and establishing a clear participation framework that respects the federated model, allowing institutions to retain control of their data while contributing to shared research value. Potential partners include a diverse and representative group with university hospitals, public institutions, large regional hospitals, research institutions, national health registries and relevant industry partners to cover clinical expertise, scientific rigour, operational capacity and technological innovation. The partnerships will be established progressively, with a clear collaboration framework, defined data-sharing agreements, technical onboarding and co-ordinated governance.

Participation will be motivated through clear incentives, which can improve research and clinical capabilities of the partners: the ability to join multinational research projects and clinical trials, access to benchmarking data within and across institutions and regions, visibility and recognition within the European healthcare ecosystem, and potential cost savings from improved efficiency, evidence-based decision-making capabilities, reduced complications and strengthened research competitiveness.

### Data model and platform

We will develop a comprehensive core data model and define a data dictionary, both designed to define standardised datasets with sufficient granularity. This includes mapping to internationally recognised clinical classifications such as SNOMED CT, LOINC, HL7 FHIR and ICD. The model should also incorporate robust quality and safety indicators—such as mortality rates, adverse event reporting, and length-of-stay metrics—to ensure analytical value across countries and different EPR systems.

Platform development focuses on creating a secure, scalable and user-friendly technology platform that manages the collection, storage, exchange and analysis of data and facilitates future real-time data streaming. Core capabilities must include encryption in transit, robust access-management controls, audit trails, automated backups, data-quality checks, interactive dashboards, feedback and benchmarking reports. To enable advanced analytics without compromising data privacy, the ETHER network will enable federated machine learning (FL). This allows collaborative model training across decentralised datasets while keeping raw patient data within local institutions. It aligns with GDPR requirements and enables large-scale, multiinstitutional research. Federated learning will support the development of integrated artificial intelligence-driven decision-support tools—such as predictive models for peri-operative complications or personalised drug dosing, while ensuring sensitive data never leaves the source.

### Data protection and compliance framework

Such a framework must be implemented to ensure that all data collection, processing and analysis comply with the General Data Protection Regulation (GDPR) and national Data Protection Acts (DPA), and sector-specific regulatory requirements. This includes privacy-by-design principles, consent and lawful-basis management and mechanisms that guarantee secure, compliant handling of sensitive health information across all participating countries.

### Implementation

A pilot implementation phase (2026 to 2028, Table 2) involves working with a limited group of healthcare institutions in selected countries to test the solution under real-world conditions. This stage validates whether the data model functions effectively from both a technical and operational standpoint, checks the feasibility and challenges in interoperability and integration with the existing EPR systems and identifies changes and refinements required before scaling up. Data collected during this phase can be used to evaluate scope, research usefulness, completeness and overall reliability. This step clarifies the types of research that the data can support, identifies gaps or potential biases, and improvements required to strengthen clinical, operational and policy insights.

### Funding strategy

The initiative is funded by ESAIC along with in-kind investments from the participating institutions during the pilot phase (2026 to 2028, Table 2). A funding model needs to be developed for future sustainability. The possibilities include leveraging EU and national research grants, forming sustainable partnerships with industry, and securing financial support from public institutions and research organisations.

### Challenges

Despite significant potential benefits, the initiative comes with a complex set of challenges that must be addressed thoughtfully to ensure long-term success and sustainability. One of the most significant challenges is navigating the GDPR, the DPAs and broader ethical requirements, including patient consent management, multinational ethical approvals, privacy laws and cross-border data-sharing agreements. Institutional policies vary widely and can introduce hurdles and delays.

The heterogeneity of data across institutions adds another layer of complexity—hospitals operate in different countries and use different EPR systems, capture data at varying levels of quality and granularity, operate in different languages and collect data on different and multiple devices and platforms, making harmonisation and standardisation very challenging. Even fundamental IT constraints, such as insufficient server security, unstable internet connectivity or a lack of appropriate devices and Synopsys Design Constraints (SDC), can limit participation. On the other hand, the ETHER network could formulate recommendations for Healthcare IT vendors to follow in order to ensure interoperability.

Human factors also play a major role. Teams may face increased workload, inadequate resources, limited training or registry fatigue, while motivation and perceived return on effort may differ across centres, influencing their readiness or willingness to share data. Additionally, many institutions struggle with translating collected data into meaningful local insights, whether for research, quality improvement, benchmarking or policy decisions.

Finally, ensuring long-term operational and financial sustainability is a major challenge, requiring a funding model and governance structure to support the platform's continued growth, maintenance and relevance.

## Conclusion

Although EPR systems have been increasingly adopted, the limited standardisation of health record databases and sharing frameworks available across Europe prohibits unlocking this huge amount of available data to improve peri-operative care. We, therefore, propose to create a European peri-operative data sharing and registry network. Establishing this ETHER network (Fig. 1) will require vision, perseverance and dedication, and collaborative investments from individuals (professionals), public entities (hospitals) and private parties (industry).
